# Prevalence and genotyping of *Chlamydia trachomatis* in symptomatic male patients from Istanbul, Turkey

**DOI:** 10.1186/s40064-016-3370-3

**Published:** 2016-10-04

**Authors:** Muammer Osman Köksal, Hayati Beka, Mehmet Demirci, Ates Kadioglu, Ali Agacfidan, Baki Akgül

**Affiliations:** 1 Department of Medical Microbiology, Istanbul Faculty of Medicine, Istanbul University, Capa, 34093 Istanbul, Turkey; 2 Department of Medical Microbiology, Cerrahpasa Faculty of Medicine, Istanbul University, Cerrahpasa, 34096 Istanbul, Turkey; 3 Department of Urology, Istanbul Faculty of Medicine, Istanbul University, 34093 Istanbul, Turkey; 4 Institute of Virology, University of Cologne, Fürst-Pückler-Str. 56, 50935 Cologne, Germany

**Keywords:** Chlamydia trachomatis, Epidemiology, Genotype distribution

## Abstract

This study was conducted to determine the prevalence and distribution of urogenital *Chlamydia trachomatis* genotypes in symptomatic male patients who were referred to the clinics of the Istanbul Faculty of Medicine. Of 419 urogenital swabs, 57 samples (13.6 %) were positive for *C. trachomatis*. Genotype distribution of *C. trachomatis*-positive samples identified five genetic variants namely genotype E as the most prevalent (36.4 %), followed by genotype G (23.6 %), H (21.8 %), D (16.4 %) and F (1.8 %). We believe that this is the first study on distribution of genotypes of *C. trachomatis* genital infections in symptomatic men in Istanbul, Turkey.

## Background

*Chlamydia trachomatis* (*C. trachomatis*) is an obligate intracellular bacterial pathogen, which continues to be the most commonly reported sexually transmitted bacterial infection and causes substantial morbidity and economic loss worldwide (WHO 2008). In 2008, the World Health Organization forecasted 106 million new urogenital chlamydia cases annually among the adult population globally. Compared to the majority of sexually transmitted infections (STIs), C*. trachomatis* infection is still the most frequently reported STI with 384.555 cases of *C. trachomatis* infection recorded in EU countries in 2013 (ECDC 2015). The prevalence of different serovars are indicators of the circulating *C. trachomatis* serovars in different geographical locations (Lysen et al. 2004). The connection between urogenital serovars and clinical manifestations is still inconclusive. While a direct correlation between clinical symptoms and certain serovars was found in some studies (Morre et al. 2000), no association was found between urogenital infections and serovars in others (Lysen et al. 2004). Currently, there are 19 identified human serovars and related variants (A, B/Ba, C, D/Da, E, F, G, Ga, H, I/Ia, J, K, L1, L2, L2a and L3) which are classified based on the antigenic variations of the major outer membrane protein (MOMP) (Morre et al. 2000). *C. trachomatis* serovars A, B/Ba and C cause conjunctival infections and are related with ocular disease. Serotypes D/Da, E, F, G, Ga, H, I/Ia, J, K cause urogenital infections, and serovars L1, L2 and L3 are responsible for lymphogranuloma venereum (LGV) (Manavi 2006). The distribution of *C. trachomatis* genotypes have been identified in several countries of the world (Lysen et al. 2004; Taheri Beni et al. 2010; Millman et al. 2004; Ngandjio et al. 2003; Gallo Vaulet et al. 2010; Yang et al. 2010) and being predominant with the genotype E (26–46 %), D (9–20 %) and F (14–24 %) in urogenital infections. Another study, in which samples were collected from countries in different geographical locations showed that *C. trachomatis* E (45 %), F (16 %), D (13 %), G (10 %) and K (%5,8) genotypes were reported as the most frequently detected genotypes in heterosexual patients (Herrmann et al. 2015).

A high resolution melting analysis (HRMA) method for cervical and urethral swabs has recently been developed for genotyping of the *omp1* gene, which encodes MOMP (Li et al. 2010).

The prevalence of genital chlamydial infection in symtomatic men in different regions of Turkey was reported in few studies and varied between 10.5 and 23 % and was found to be at rates comparable to those of European countries (Agacfidan et al. 2001; Aslan et al. 2002; Kaleli et al. 1999). But so far, no data exists on the circulating genotype distribution in Turkey. The aim of this study was to determine and characterize the prevalence of *C. trachomatis* genotypes on symptomatic male patients attending a STD clinic in Istanbul University Faculty of Medicine by HRMA and sanger sequencing.

## Methods

### Patients

A total of 419 urogenital swab specimen were collected from male patients (aged 18–68) with symptoms (discharge, pain during urination, genito-urinary pain during sexual intercourse) for the investigation of STDs. Written informed consent for participation in this study was obtained from all participants. The authors assert that all procedures contributing to this work were approved by the Ethics Committee of Istanbul University (reference number 2015/1244), comply with the ethical standards on human experimentation and with the Helsinki Declaration of 1975, as revised in 2008. The swabs were placed in medium for *Chlamydia* PCR and were stored at 4 °C until transportation to the laboratory and then stored at −20 °C until real time PCR for *C. trachomatis* DNA was performed.

### Real time PCR analysis of *C.trachomatis* DNA

DNA was prepared from urogenital samples using high pure PCR template preparation kit (Roche Diagnostics GmBH, Mannheim Germany) and suspended in 200 µl elution buffer according to the manufacturer’s protocols (Morre et al. 1998).After quantification of isolated DNA, probes were studied according to the manufacturer’s instructions on Light Cycler 2.0 (Roche Diagnostics GmBH, Mannheim Germany) instrument. The master mix for *C. trachomatis* DNA real time PCR reactions were prepared using LightCycler^®^ Fast Start DNA Master HybProbe kit (Roche Diagnostics GmBH, Mannheim, Germany) and Lightmix *C. trachomatis* kit (TIB Molbiol GmBH, Berlin, Germany). 5 µl of template DNA were added to each capillary tube for a final reaction volume of 20 µl. One negative and one positive control were included in each run. The PCR profile was an initial denaturation of template and activation of enzyme at 95 °C for 10 min followed by 50 cycles of 95 °C for 5 s, 62 °C for 5 s, and 72 °C for 15 s. Data analysis was performed as described in the LightCycler instrument operator’s manual. The criteria used for determining positive and negative samples depended on the amplification curves at a wavelength of 640 nm. *C.trachomatis* positive DNAs and samples were stored at −70 °C for further analysis.

### HRM analysis

The external primers for the VS1–VS2 PCR were CT1: 5′-TGA ACC AAG CCT TAT GAT CGA CGG A-3′ and CT2: 5′-CGG AAT TGT GCA TTT ACG TGA G-3′. The internal PCR primers were selected as previously described by Zheng et al. (2007) and were CT3: 5′-ACT TTG TTT TCG ACC GTG TTT TG-3′ and CT4: 5′-GAT TGA GCG TATTGG AAA GAA GC-3′. All primers were synthesized from IDT (Integrated DNA Technologies, Coraville, Iowa). *C.trachomatis* positive DNA samples were amplified in 96-well plates. LightCycler 480 High Resolution Master kit (Roche Diagnostics GmBH, Mannheim Germany) was used for HRM analysis in a LightCycler 480 II system (Roche Diagnostics GmBH, Mannheim Germany). In brief, each reaction was performed in a final volume of 10 μl containing 20 ng of DNA, 0.25 mM of each primer (forward or reverse) and 1 × LightCycler 480 HRM Master mix (Roche). The PCR profile was: an initial denaturation of template and activation of enzyme at 95 °C for 10 min followed by 40 cycles of 95 °C for 10 s, 57 °C for 10 s, and 72 °C for 20 s. At the end of the PCR cycles, PCR products were denatured at 95 °C for 1 min and rapidly cooled to 40 °C. HRM analyses were performed from 72 °C to 95 °C at a temperature gradient of 1 °C/sec, acquiring 25 data points per °C. Each sample was run in duplicate for analysis. All samples were grouped by the LightCycler 480 Gene Scanning Software.

### Sequencing of the omp1 gene

DNA from *C. trachomatis*-positive samples was purified using the Qiaquick PCR Purification Kit (Qiagen, Hilden, Germany) as instructed by the manufacturer. The omp1 gene was sequenced using the Big Dye Sequencing Terminator Kit and ABI 3130×l sequencing system (Applied Biosystems, Foster City, CA, USA) with HRMA VS-1 and VS-2 primers. The strains with corresponding accession numbers B/IU-1226 (AF063208), B/B-16 (AY950630), D/B-120 (X62918), Da/TW-448 (X62921), E/Bour (X52557), F/ICCal3 (X52080), G/UW57 (AF063199), H/Wash (H/UW4) (X16007), I/UW12 (AF063200),J/UW36 (AF063202) and K/UW31 (AF063204) were used as reference sequences. The omp1 sequences were edited, aligned, and analysed using the MEGA6 software.

### Statistical analysis

Statistical analysis was performed using the Chi square test and IBM SPSS version 20 software (IBM Corporation, Armonk, NY, USA) for establish relationship between *C.trachomatis* prevalence and patient’s age.

## Results and discussion

A total of 419 urogenital swab specimen from symptomatic male patients were studied for *C. trachomatis*. 57 out of these 419 samples (13.6 %) were DNA positive for *C. trachomatis* (Table 1). This prevalence rate of *C. trachomatis* in symptomatic men lies within the previously detected range in other turkish epidemiological studies (Agacfidan et al. 2001; Aslan et al. 2002; Kaleli et al. 1999). The largest proportion of cases in European countries reported in 2013 were among young people between 20 and 24 of age who accounted for 41 % of cases. The second largest group was the age group of 15–19 years old accounting for 26 % (ECDC 2015). However, in comparison to the age-related infection rates of European countries with the highest *Chlamydia* prevalence among persons with 15–24 of age, rates in our study were found to peak in the age group 20–29 (χ^2^ = 35,587, p < 0,001). The reason is suggested to be the older age at onset of sexual activity in Turkey when compared to European countries.

**Table 1:** Prevalence of cervical *C. trachomatis* infection in different age groups as determined by real-time PCR

Age group	<20	20–24	25–29	30–34	35–44	+45	
PCR +	2 (50 %)	17 (34 %)	17 (15.9 %)	12 (16.4 %)	7 (6.1 %)	2 (2.9 %)	57 (13.6 %)
PCR -	2 (50 %)	33 (66 %)	90 (84.1 %)	61 (83.6 %)	108 (93.9 %)	68 (97.1 %)	362 (86.4 %)
Total	4	50	107	73	115	70	419

**Fig. 1:**
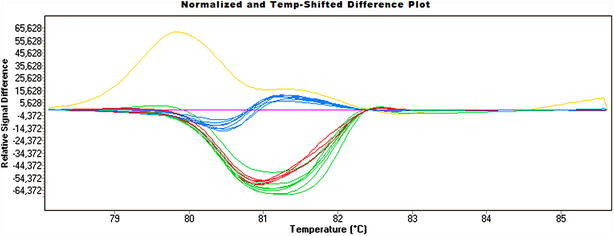
Identification of five different STI-related *C. trachomatis* genotypes by gene scanning and genotyping analysis. Genotype specific curves were distinguishable and could be differentiated through direct visualization (*green* genotype E, *red* genotype D; *blue* genotype G, *pink* genotype H, *yellow* genotype F)

The genotypes of the 57 *C. trachomatis*-positive samples were analysed by HRMA and a total of 55 omp1 fragments could successfully be amplified. After the HRM group analysis we discriminated 5 genotype clusters in our materials (Fig. 1) and chose two different samples from each genotypes cluster for sequencing of the omp1 gene. The five *C. trachomatis* genotypes were identified to be genotype E (n = 20, 36.4 %) as the most prevalent, followed by G (n = 13, 23.6 %), H (n = 12, 21.8 %), D (n = 9, 16.4 %) and F (n = 1, 1.8 %) (Table 2). The predominance of genotype E is in line with previous studies on different patient groups (Taheri Beni et al. 2010, Ngandjio et al. 2003, Gallo Vaulet et al. 2010, Mejuto et al. 2013). Genotypes E, F and D are known to be the most prevalent genotypes circulating in heterosexual populations worldwide (Lysen et al. 2004; Taheri Beni et al. 2010; Millman et al. 2004; Ngandjio et al. 2003; Gallo Vaulet et al. 2010; Yang et al. 2010). An unusual finding of this study was the relatively high prevalence (23.6 %) of genotype G in our cohort. This genotype has widely been reported for the population of men having sex with men in European and other countries (Li et al. 2011; Mejuto et al. 2013; Quint et al. 2011; Twin et al. 2011). The high genotype G rates in our study might suggest a high number of men among the analyzed patients who have sex with men. Genotype H showed the highest frequency (72.7 %) in male patients in the age group 30–34. Interestingly, *C.trachomatis* genotypes J,K and I/Ia found in male patients in other reports (Lysen et al. 2004; Millman et al. 2004; Herrmann et al. 2015), were not detected in our study, which may indicate a specific geographical distribution of certain genotypes. On the other hand the number of samples included in the analysis was relatively small and genotypes with low prevalence rates might have been missed.

**Table 2:** Distribution of cervical *C. trachomatis* genotypes in relation with age

Genotypes	15–19	20–24	25–29	30–34	35–44	+45	Total
D	1 (50 %)	3 (18.8 %)	5 (29.4 %)	–	–	–	9 (16.4 %)
E		6 (37.5 %)	7 (41.2 %)	2 (18.2 %)	4 (57.1 %)	1 (50 %)	20 (36.4 %)
F	–	–	–	–	1 (14.3 %)		1 (1.8 %)
G	1 (50 %)	5 (31.2 %)	4 (23.5 %)	1 (9.1 %)	1 (14.3 %)	1 (50 %)	13 (23.6 %)
H	–	2 (12.5 %)	1 (5.9 %)	8 (72.7 %)	1 (14.3 %)	–	12 (21.8 %)
Total	2	16	17	11	7	2	55

In conclusion, we detected five different *C.trachomatis* genotypes among our patients. This diversity might be associated with sexual behavior among different religious and ethnic groups living in Istanbul. We believe that this is the first study investigating the prevalence and distribution of genotypes of *C. trachomatis* genital infections of symptomatic men in Istanbul, Turkey.
